# The Type VI Secretion System Encoded in SPI-6 Plays a Role in Gastrointestinal Colonization and Systemic Spread of *Salmonella enterica* serovar Typhimurium in the Chicken

**DOI:** 10.1371/journal.pone.0063917

**Published:** 2013-05-14

**Authors:** David Pezoa, Hee-Jeong Yang, Carlos J. Blondel, Carlos A. Santiviago, Helene L. Andrews-Polymenis, Inés Contreras

**Affiliations:** 1 Departamento de Bioquímica y Biología Molecular, Facultad de Ciencias Químicas y Farmacéuticas, Universidad de Chile, Santiago, Chile; 2 Department of Microbial and Molecular Pathogenesis, Texas A&M University System Health Science Center, College of Medicine, College Station, Texas, United States of America; Indian Institute of Science, India

## Abstract

The role of the *Salmonella* Pathogenicity Islands (SPIs) in pathogenesis of *Salmonella enterica* Typhimurium infection in the chicken is poorly studied, while many studies have been completed in murine models. The Type VI Secretion System (T6SS) is a recently described protein secretion system in Gram-negative bacteria. The genus *Salmonella* contains five phylogenetically distinct T6SS encoded in differentially distributed genomic islands. *S.* Typhimurium harbors a T6SS encoded in SPI-6 (T6SS_SPI-6_), which contributes to the ability of *Salmonella* to colonize mice. On the other hand, serotype Gallinarum harbors a T6SS encoded in SPI-19 (T6SS_SPI-19_) that is required for colonization of chicks. In this work, we investigated the role of T6SS_SPI-6_ in infection of chicks by *S.* Typhimurium. Oral infection of White Leghorn chicks showed that a ΔT6SS_SPI-6_ mutant had reduced colonization of the gut and internal organs, compared with the wild-type strain. Transfer of the intact T6SS_SPI-6_ gene cluster into the T6SS mutant restored bacterial colonization. In addition, our results showed that transfer of T6SS_SPI-19_ from *S*. Gallinarum to the ΔT6SS_SPI-6_ mutant of *S.* Typhimurium not only complemented the colonization defect but also resulted in a transient increase in the colonization of the cecum and ileum of chicks at days 1 and 3 post-infection. Our data indicates that T6SS_SPI-6_ contributes to chicken colonization and suggests that both T6SS_SPI-6_ and T6SS_SPI-19_ perform similar functions *in vivo* despite belonging to different phylogenetic families.

## Introduction

Nontyphoidal *Salmonella* gastroenteritis has an estimated global burden of 93.8 million cases per year, of which 80.3 million cases are likely to be food-borne [1]. The most prevalent serovars responsible for food-borne salmonellosis are *S. enterica* serovar Enteritidis and *S. enterica* serovar Typhimurium [2]. *Salmonella enterica* serovar Typhimurium (*S*. Typhimurium) is a broad host-range pathogen able to infect humans, mice and birds. In mice, this serovar causes a systemic infection similar to human typhoid fever that results from infection with serovar Typhi (as well as Paratyphi A, B, and C) [3], [4]; for this reason the murine model has been widely used to study the pathogenesis of *Salmonella* infection. In humans however, *S.* Typhimurium causes self-limiting gastroenteritis characterized by abdominal pain, vomiting and inflammatory diarrhea [5]. In contrast, this pathogen is able to colonize the chicken without clinical symptoms, and is thus a major vehicle for transmission of salmonellosis to humans.

Studies conducted using murine models of infection and *in vitro* cell culture systems have identified numerous genes required to establish a successful infection by *S.* Typhimurium. Most genes are clustered in genomic islands known as *Salmonella* Pathogenicity Islands (SPIs) [6]–[10]. Of the five SPIs (SPI-1 to SPI-5) common to all serovars of *Salmonella enterica*, the SPI-1 and SPI-2 are the two major virulence determinants of *Salmonella.* Each of these SPIs encodes two different type III secretion systems (T3SS) that deliver effector proteins directly into the cytoplasm of eukaryotic cells [11], [12]. The T3SS_SPI-1_ is mainly involved in invasion of intestinal epithelial cells [13], [14] but it is also required for intracellular proliferation and for the biogenesis of the *Salmonella* containing vacuole inside infected cells [15], [16]. The T3SS_SPI-2_ is essential for survival within phagocytic cells and systemic infection [17].

Studies on the role of the SPIs in the pathogenesis of *S.* Typhimurium infection in the chicken are few and are sometimes contradictory. While some authors reported that both T3SS_SPI-1_ and T3SS_SPI-2_ are required for the infection process [18]–[21], one study showed that neither T3SS_SPI-1_ nor T3SS_SPI-2_ is critical for colonization of chickens [9]. One report directly compared the intestinal and systemic colonization of *Salmonella-*resistant mice and one-week-old chickens by *S.* Typhimurium [22]. Infected chicks had very few organisms in internal organs and no symptoms of systemic effects, while in mice, spleen and liver were colonized by bacteria and showed significant enlargement. Furthermore, colonization of the intestine had a different dynamic in the chicken versus the mice models of infection, as SPI-1 was important for association to the intestinal epithelium of the chicken rather than for invasion, as is the case in mice [22]. From these studies, it is evident that the murine model has a limited applicability to *Salmonella* infection of the chicken, and that genes in addition to the highly conserved SPIs are required for chicken colonization and systemic spread.

Type VI secretion systems participate in a variety of different processes, ranging from inter-bacterial relationships to pathogenesis [23]–[27]. Gram-negative bacteria carrying T6SS clusters include human, animal and plant pathogens [28]–[34]. The genus *Salmonella* contains five phylogenetically distinct T6SS loci; four of them are differentially distributed among serovars of *S. enterica*, while the fifth T6SS is present in *S. bongori* [35], [36]. Two of these clusters, T6SS_SPI-6_ and T6SS_SPI-19_, have been linked to *Salmonella* pathogenesis. T6SS_SPI-6_ is required for intracellular replication in macrophages and systemic dissemination in mice by *S*. Typhimurium [37]–[41] and *S.* Typhi [29], while T6SS_SPI-19_ contributes to colonization of the gastrointestinal tract and internal organs of chickens by *S.* Gallinarum strain 287/1 [42].

In this study we have investigated the contribution of T6SS_SPI-6_ to *S.* Typhimurium ability to colonize the gastrointestinal tract and internal organs of White Leghorn chicks. We have also addressed whether T6SS_SPI-19_ of *S.* Gallinarum can rescue the colonization defect of a *S*. Typhimurium mutant lacking T6SS_SPI-6_. Through competitive index experiments we demonstrate that T6SS_SPI-6_ is crucial to gastrointestinal colonization and systemic spread of *S.* Typhimurium in chicks. In addition, we show that transfer of T6SS_SPI-19_ restores the colonization defect of a mutant lacking T6SS_SPI-6_, indicating that both T6SS perform similar functions *in vivo* despite belonging to different phylogenetic families.

## Materials and Methods

### Bacteria and Growth Conditions

The bacterial strains used in this work are listed in **Table 1**. Bacteria were routinely cultivated in LB broth (10 g/l tryptone, 5 g/l yeast extract, 5 g/l NaCl) at 37°C with aeration or on LB plates (15 g/l agar) supplemented with the appropriate antibiotic at the following concentrations: Ampicillin (Amp), 100 µg/ml; Kanamycin (Kan), 50 µg/ml; Chloramphenicol (Cam), 20 µg/ml; Trimethoprim (Tm), 100 µg/ml; Spectinomycin (Sp), 250 µg/ml.

**Table 1:** Strains and plasmids used in this study.

Strains	Features	Source of reference
***Escherichia coli***		
DH5α	F^-^Φ80*lacZ*ΔM15Δ(*lacZYA-argF*)U169 *deoR recA1 endA1 hsdR17*(r_k_^-^, m_k_^+^) *phoA* *supE44 thi-1 gyrA96 relA1* λ^-^	Laboratory collection
EC100D *pir-116*	*F^-^mcrAΔ(mrr-hsdRMS-mcrBC)*Φ *80dlacZΔM15 ΔlacX74 recA1 endA1 araD139* *Δ(ara, leu)7697 galU galK λ- rpsL (Str^R^) nupG pir-116(DHFR)*	Laboratory collection
EC100D *pir-116*/R995+SPI-6	Strain with T6SS_SPI-6_ from *S*. Typhimurium cloned in plasmid R995	This study
EC100D *pir-116*/R995+SPI-19	Strain with T6SS_SPI-19_ from *S*. Gallinarum cloned in plasmid R995	[42]
DH5α/R995	Strain harboring an empty R995 vector	This study
DH5α/R995-VC6	Strain containing a derivative of plasmid R995 with a 1,209 bp DNAfragment of T6SS_SPI-6_ cloned from *S*. Typhimurium	This study
***Salmonella*** ** Typhimurium**		
14028****s	Wild-type strain	Laboratory collection
MTM753	14028****s Δ*phoN*	This study
MTM35	14028****s ΔSPI-6 T6SS	This study
MTM2640	14028****s Δ*clpV*	This study
WT/R995	14028****s containing an empty R995 vector	This study
MTM35R	MTM35 harboring R995 plasmid	This study
MTM35R6	MTM35 complemented with plasmid R995+SPI-6	This study
MTM35R19	MTM35 complemented with plasmid R995+SPI-19	This study
**Plasmids**		
pKD46	*bla* P_BAD_ *bet exo* pSC101 oriT^s^, Amp^R^	[43]
pEKA30	IncQ plasmid that constitutively express Cre recombinase, Amp^R^	[44]
pCLF2	Red-swap redesigned vector, Cam^R^	[50]
pCLF4	Red-swap redesigned vector, Kan^R^	[50]
pVEX1212	Suicide vector harboring a *loxP* site followed by a Sp^R^ cassette	[44]
pVEX2212	Suicide vector harboring a *loxP* site followed by a Cam^R^ cassette	[44]
R995	Self-transmissible broad-host range IncP vector	[44]
R995-VC6	A derivative of plasmid R995 with a 1,209 bp DNA fragment of T6SS_SPI-6_cloned from *S*. Typhimurium	This study
R995+SPI-6	T6SS_SPI-6_ cluster from *S*. Typhimurium 14028****s cloned in vector R995	This study
R995+SPI-19	T6SS_SPI-19_ cluster from *S*. Gallinarum 287/91 cloned in vector R995	[42]

### DNA Methods

DNA manipulations were performed using standard protocols. Plasmid DNA was isolated from overnight cultures using the QIAprep Spin Miniprep Kit (QIAGEN), according to the manufactureŕs instructions. Genomic DNA was isolated from overnight cultures utilizing the GenElute Bacterial Genomic DNA kit (Sigma) according to the manufactureŕs instructions. PCR products were purified using the QIAquick PCR Purification Kit (QIAGEN). *Xba*I restriction enzyme (Fermentas) and T4 DNA ligase (New England Biolabs) were used as per manufacturer instructions. DNA samples were routinely analyzed by electrophoresis in 1% agarose gels (1X Tris-acetate-EDTA buffer) and visualized under UV light after ethidium bromide staining.

### PCR Amplifications

Primers were designed using the Vector NTI Advance 10.0 software (Invitrogen) and are listed in **Table 2**. PCR amplifications were performed in a MultiGene TC9600-G thermal cycler (LabNet), using GoTaq Flexi DNA Polymerase (Promega). Conditions for tiling-PCR amplification were as follows: 3 min at 94°C followed by 30 cycles of incubations at 94°C for 30 s, 58°C for 30 s, and 72°C for 4 min, followed by a final extension step at 72°C for 7 min. Conditions for standard PCR amplification were as follows: 3 min at 94°C followed by 30 cycles of incubations at 94°C for 30 s, 55°C for 30 s, and 72°C for 2 min, followed by a final extension step at 72°C for 5 min. When required, PCR products were purified by using the QIAquick PCR purification kit (Qiagen).

**Table 2:** Primers used in this study.

Primer	Sequence^a^
**Mutagenesis**	
SPI-6_T6SS_(H1+P1)	AGGGTGTTTTTATACATCCTGTGAAGTAAAAAAAACCGTA*GTGTAGGCTGGAGCTGCTTC*
SPI-6_T6SS_(H2+P2)	GTGAACATGGCACATTAATTTGAAGCAGCTCTCATCCGGT*CATATGAATATCCTCCTTAG*
SPI-6_OUT5	CCGAAGTGTATCTGGCGATGA
STM0272_(H1+P1)	GGCATAACACATGGAAACTCCTGTTTCACGCAGTGCGTTG*GTGTAGGCTGGAGC TGCTTC*
STM0272_(H2+P2)	ACGGCCGGTTTCAGCAAACGATCTCAAAAACAATCTGCTC*CATATGAATATCCTCCTTAG*
STM0272_OUT5	GGCGGCAGTAAATACGATGT
STM_Δ*phoN*_(H1+P1)	GTGAGTCTTTATGAAAAGTCGTTATTTAGTATTTTTTCTA*GTGTAGGCTGGAGCTGCTTC*
STM_Δ*phoN*_(H2+P2)	ACTTTCACCTTCAGTAATTAAGTTCGGGGTGATCTTCTTT*CATATGAATATCCTCCTTAG*
STM_Δ*phoN*_OUT5	TTGCCTGATCCGGAGTGA
K1	CAGTCATAGCCGAATAGCCT
C3	CAGCTGAACGGTCTGGTTATAGG
**VEX Capture**	
STM0266_VEX_H1_U1	*GGCCACGTGGGCCGTGCACCTTAAGCTT*
STM0266_VEX_H2_U2	GAGGTTATTCATGTCAACAGGATTACGTTTCACACTGGA*GGTGCAGGCTGGAGCTGCTTC*
STM0298_VEX_H1_D1	GGGGAGGTTGTGCGACGTTTGCATAATCCAGCAAGAACTG*GGTTTAACGGTTGTGGACAACAAGCCAGGG*
STM0298_VEX_H2_D2	ACACAGGCCAGACTGATTATACAGGCATGAAAAAGCTCTC*CAGGTCGACGTCCCATGGCCATTCGAATTC*
STM_VC_OUT5	GCTCTAGACCGGAGGGGTTATCTTTTCC
STM_VC_OUT3	GCTCTAGATTGAAGCAGCTCTCATCCGG
5trfA	ACGTCCTTGTTGACGTGGAAAATGACCTTG
3trfA	CCGGAAGGCATACAGGCAAGAACTGATCG
SPI-6_OUT_DOWN	AAACGGGTCTATTTACAGGGGCAC
**Tiling-PCR**	
1_T6SS_SPI-6_FOR	TTCAAGAAGTTCCACCGTCTATCG
1_T6SS_SPI-6_REV	ACCTGTTTGAGCTGCTACATACCAG
2_T6SS_SPI-6_FOR	CATTCAGTTCGCCGTCAAAGTG
2_T6SS_SPI-6_REV	CCGCTGCGAATTTTGTTATCG
3_T6SS_SPI-6_FOR	CCACGTTCTTCGGCATTACCAG
3_T6SS_SPI-6_REV	CGGTGTTGTAAACCAGATGCTCC
4_T6SS_SPI-6_FOR	AGACGCTGGCGAACACGATC
4_T6SS_SPI-6_REV	TAAGCACTGGCCGTAGCTCTGG
5_T6SS_SPI-6_FOR	GCAGCCATCCTTTGCACAAG
5_T6SS_SPI-6_REV	GGTTGTGTTATTGGCGGCTTC
6_T6SS_SPI-6_FOR	TATGCGATCAGGCGAACCTG
6_T6SS_SPI-6_REV	TCTTCCTGTAACCGGGTATCCAG
7_T6SS_SPI-6_FOR	GGTTGGATCAGGGACTGGATACC
7_T6SS_SPI-6_REV	CGTAACCCTCAACATCCTGCG
8_T6SS_SPI-6_FOR	AAAGCACCGGTGAATGTGGCTG
8_T6SS_SPI-6_REV	TCGGTGTGGTCATCCTTACGGG
9_T6SS_SPI-6_FOR	TGTCAGCACCAACAGTCGCC
9_T6SS_SPI-6_REV	CGCCCTTCGATAGAATCTGGC
10_T6SS_SPI-6_FOR	TAGTAGGGCCAGATTCTATCGAAGG
10_T6SS_SPI-6_REV	CCCTCCGGCTTTTACACATTATTC

a Italics indicate the region that anneals to the 5′ or 3′ end of the antibiotic resistance cassette used for the mutagenesis. Underline indicates *XbaI* restriction sites used for cloning an internal region of homology to T6SS of SPI-6 into R995 plasmid.

### Construction of *S*. Typhimurium Mutant Strains

Mutants of *S*. Typhimurium carrying deletions of the T6SS_SPI-6_ gene cluster and the *clpV* (STM0272) or *phoN* genes were constructed using the Lambda-Red System [43]. The oligonucleotides used for the mutagenesis are shown in **Table 2** and the sequences of plasmids pCLF2 and pCLF4 used as templates are available in GenBank (accession numbers HM047089 and EU629214.1, respectively). The correct insertion of the resistance cassettes was checked by PCR, and confirmed mutations were moved to a clean genetic background by generalized transduction using the high-frequency transducing phage P22 HT105/1 *int*-201. To be able to identify wild type versus mutant colonies in the mixed competition experiments, the *S*. Typhimurium Δ*phoN* mutant was used as the wild type strain. *phoN+* and *phoN-* strains can be distinguished by blue-white selection on 5-bromo-4-chloro-3-indolyl phosphate (XP) containing media, *phoN-* strains form white colonies while *phoN+* strains appear blue. Mutations in *phoN* do not affect the ability of *S*. Typhimurium to colonize and persist in the chick [22].

### Cloning of *S*. Typhimurium SPI-6 by VEX-Capture

Cloning of a ∼39 Kb fragment containing the T6SS_SPI-6_ gene cluster from *S*. Typhimurium 14028s onto plasmid R995 was performed by the VEX-Capture technique for the targeted excision and cloning of large DNA fragments [44]. First, *loxP* sites were inserted at each side of the targeted genomic region by homologous recombination of PCR products by the Lambda-Red system, using as templates the plasmids pVEX1212 and pVEX2212 that encode Sp and Cam resistance cassettes, respectively. Correct insertion of *loxP* sites was confirmed by PCR using primers SPI-6_OUT5 and STM0266_VEX_H2_U2 for *loxP* insertion located in the upstream region of the T6SS cluster, and primers SPI-6_OUT_DOWN and STM0298_VEX_H2_D2 for the downstream *loxP* insertion. This cluster was excised from the chromosome as a non-replicating circular DNA molecule by specific recombination of *loxP* sites mediated by the action of Cre recombinase encoded in plasmid pEKA30. This intermediate was captured into the R995-VC6 vector by a homologous recombination event, producing the R995+SPI-6 plasmid. The R995-VC6 plasmid contains a 1,209 bp internal region of homology to the T6SS_SPI-6_ cluster, cloned by PCR amplification with primers STM_VC_OUT5 and STM_VC_OUT3 (**Table 2**).

Plasmid R995+SPI-6 was transferred to *E. coli* strain EC100D *pir-116* by conjugation and the presence and structural integrity of the T6SS_SPI-6_ gene cluster cloned onto R995 was verified by tiling-PCR analysis in order to amplify ten fragments that cover the entire T6SS region (**Figure S1**). For competitive infections in chickens, the *in vivo* stability of plasmids R995 and R995+SPI-6 was assessed in each organ at each time point studied. No differences were observed on colony forming units (CFU) indicating that R995 and its derivatives are highly stable *in vivo*.

### Experimental Infections of Chickens

*S.* Typhimurium strains were grown aerobically at 42°C for 16 hours in LB broth. This temperature of incubation was used because it corresponds to the body temperature of chicks. For single and competitive infections, fifteen 4-day old unsexed White Leghorn chicks were orally inoculated with 10^9^ CFU of a single strain or with an equal mixture of the strains to be tested in a volume of 100 µl of sterile PBS. The inoculum was serially diluted and plated to determine the titer and input ratio. Five birds from the infected group were sacrificed by asphyxiation with CO_2_ on days 1, 3 and 9 post-infection. Ileum, cecum (including contents), liver and spleen were collected. These organs were homogenized in sterile PBS and serial ten-fold dilutions spread on LB agar plates containing the appropriate antibiotics for determination of CFU. For histopathological analysis, the cecum and liver of experimental animals were fixed in 10% formalin for 24 h followed by incubation in 70% ethanol and then embedded in paraffin. The samples were stained with hematoxylin and eosin and 10 fields per sample were examined and scored by a trained veterinary pathologist to determine histopathological changes.

### Statistical Analysis

Data obtained from competitive infection experiments were calculated as a mean ratio of logarithmically converted CFU of mutant to wild type normalized to the input ratio. Error bars indicate standard error. Statistical significance was determined using a two-tailed Students *t*-test. *P* values of <0.05 were considered statistically significant (SPSS software, SPSS, Inc., Chicago, IL).

### Ethics Statement

All animal experiments in this study were approved by the Texas A&M University Institutional Animal Care and Use Committee (TAMU AUP# 2010-38) and were carried out in accordance with the Guide to the Care and Use of Laboratory Animals, the Public Health Service Policy on the Human Care and Use of Laboratory Animals.

## Results

### The T6SS Encoded in SPI-6 Contributes to Efficient Colonization of the Avian Host by *Salmonella* Typhimurium

Single infections and competitive index experiments were performed to determine the contribution of the SPI-6 T6SS to intestinal and systemic colonization of chicks by *S*. Typhimurium.

**Figure 1:**
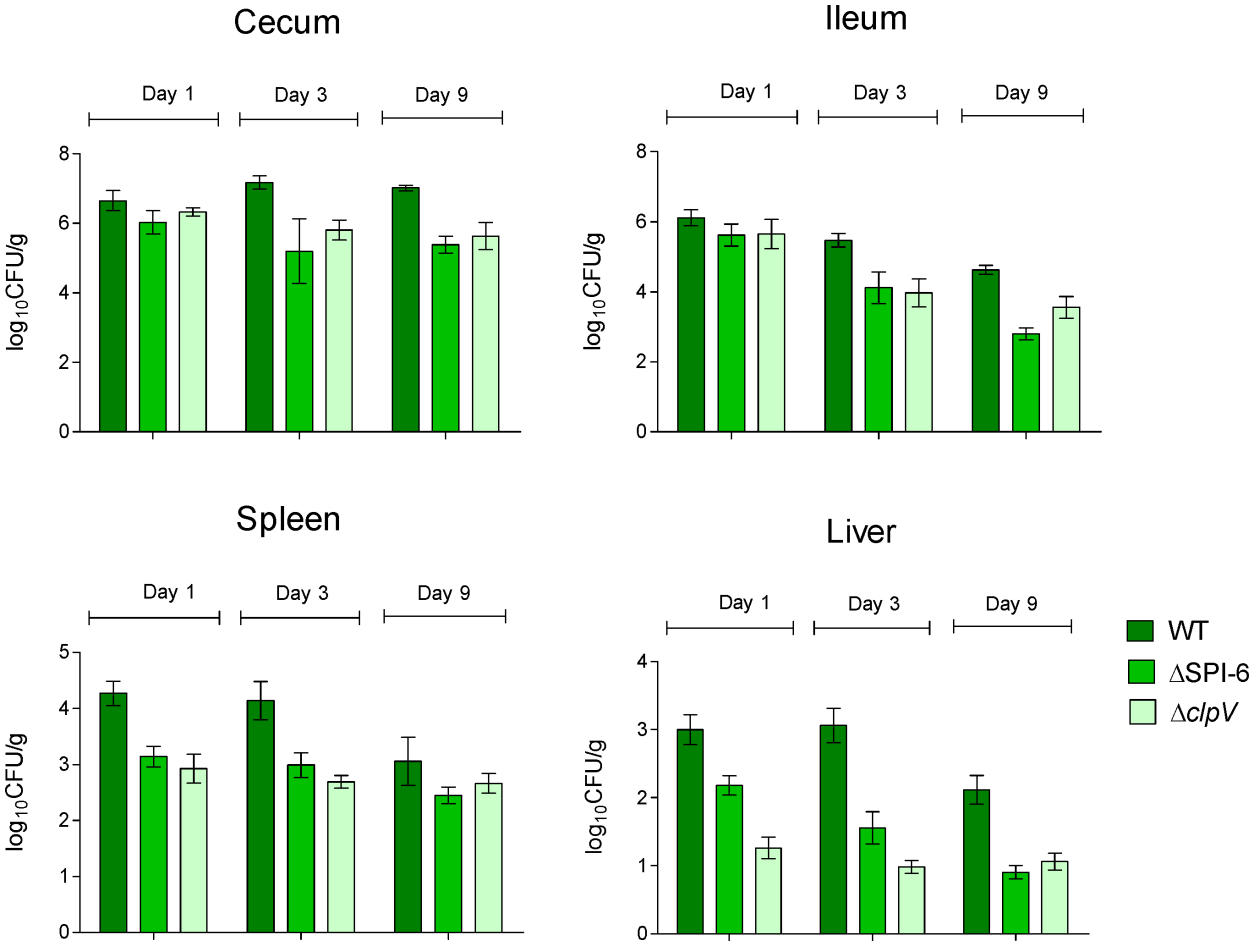
Distribution of *S.* Typhimurium 14028 s and SPI-6 T6SS mutants in the gastrointestinal tract and internal organs of orally infected chickens. Four-day-old White Leghorn chicks were infected by gavage with 10^9^ CFU of either the wild-type *S*. Typhimurium 14028****s, the ΔT6SS_SPI-6_ mutant or the Δ*clpV* mutant strains. After 1, 3 and 9 days post-infection, the chicks were humanely euthanized and the ileum, cecum, liver and spleen were aseptically removed. Tissues were homogenized and viable bacterial counts were determined. Data are mean values of log_10_ CFU/g of tissue, from five animals at each time point.

For single infections, White Leghorn chicks were orally-infected with either the wild-type strain, a ΔT6SS_SPI-6_ mutant (MTM35) or a Δ*clpV* deletion mutant (MTM2640) and colonization of the cecum, ileum, liver and spleen was evaluated over 9 days of infection. ClpV, a conserved structural component of the T6SS that belongs to Clp/Hsp100 AAA+ of ATPase superfamily, is required for the activity of the secretion system [45], [46]. As shown in **Figure 1**, the cecum and ileum of chicks infected with the wild-type strain were heavily colonized at all time points, while the liver and spleen were only lightly colonized, as reported previously [22]. Interestingly, both the ΔT6SS_SPI-6_ and Δ*clpV* mutant strains showed an overall lower degree of colonization of the cecum and ileum from day 3 post-infection and of the liver and spleen from day one post-infection, suggesting a role for the SPI-6 T6SS in chick colonization.

**Figure 2:**
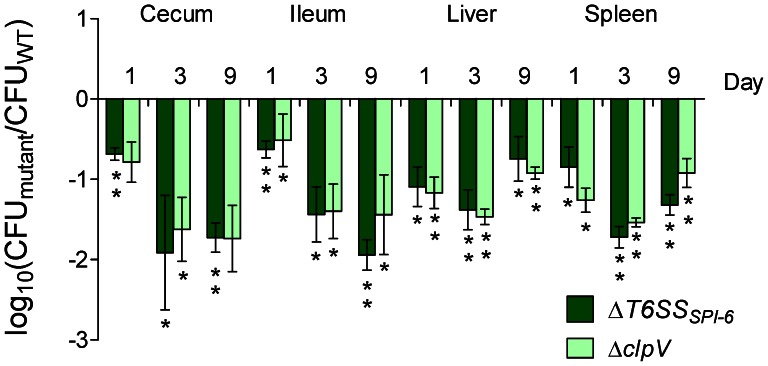
*In vivo* competition between ΔT6SS_SPI-6_ and Δ*clpV* deletion mutants and the wild type *S*. Typhimurium strain 14028 s. Fifteen four-day-old White Leghorn chicks were infected intragastrically by gavage with 10^9^ CFU of a mixture at a 1∶1 ratio of the respective mutant strain and the wild type *S*. Typhimurium 14028****s. At 1, 3 and 9 days post-infection groups of 5 chicks were sacrificed and organs were excised, homogenized, and serially diluted to determine bacterial loads. Bars represent the geometric mean of the log ratio of the mutant CFU/wild type CFU, normalized to the inoculum ratio. Error bars denote standard error. Statistical significance was determined using a two-tailed Student’s *t* test, and asterisks indicate that normalized output ratios were significantly statistically different from the equivalent ratio in the inoculum (*P<0.05; **P<0.001).

In order to determine the competitive fitness within the host, of each mutant strain, competitive index experiments were performed. White leghorn chicks were orally infected with a mixture of each mutant with the wild-type strain at a 1∶1 ratio and colonization of each organ was evaluated over 9 days of infection. As shown in **Figure 2**, a strong colonization defect was observed for both the ΔT6SS_SPI-6_ and Δ*clpV* mutants during intestinal and systemic colonization from day 1 post-infection. This markedly attenuated phenotype was more pronounced at the third day post-infection and it was maintained throughout day 9 in each organ analyzed. These results indicate that *S*. Typhimurium requires a functional T6SS to efficiently colonize the avian host.

**Figure 3:**
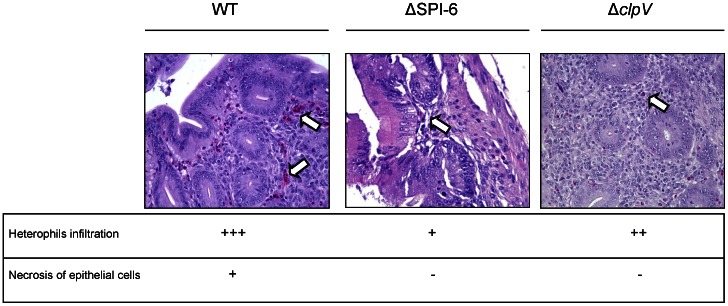
Histopathological changes in the cecum of infected chicks at day 3 post-infection. Groups of 3 White Leghorn chicks were inoculated intragastrically by gavage with 10^9^ CFU of the wild type *S*. Typhimurium 14028****s strain, the ΔT6SS_SPI-6_ mutant strain or the Δ*clpV* mutant strain. At day 3 post-infection the chicks were sacrificed and the ceca were excised, fixed, stained with hematoxylin and eosin, and analyzed for histopathological lesions. Representative images of stained sections (400X) and scores for histopathological lesions in the cecum of infected chicks are shown (-, no changes; +, mild; ++, strong; +++, severe). White arrows indicate heterophil infiltration.

Histopathological analysis of the cecum and liver from infected birds was performed to determine whether or not this attenuated phenotype was accompanied by tissue damage and/or signs of an inflammatory response. Single infections were performed as described above, and 3 days post infection the chicks were sacrificed and each organ tested was excised, fixed, stained with hematoxylin and eosin, and analyzed for histopathological lesions. Significant pathological changes were observed in the cecum of chicks infected with the wild-type strain. Among these changes, focal necrosis of the mucosal epithelial cells and heterophil infiltration were evident, indicating a strong inflammatory response induced by *S.* Typhimurium 14028****s (**Figure 3**, left panel). In contrast, chicks infected with either the ΔT6SS_SPI-6_ or Δ*clpV* mutant strains showed a considerable lower level of heterophil infiltration in the cecum, with no signs of necrosis of the epithelial cells (**Figure 3**, central and right panels, respectively). No significant histopathological differences were found in livers infected with either the wild-type or the T6SS mutants (data not shown). Absence of lesions in the liver are most probably due to the low levels of bacterial colonization of internal organs by both the wild-type and T6SS mutant strains (**Figure 1**).

### The Colonization Defect of the ΔT6SS_SPI-6_ Mutant is Complemented by Transfer of the T6SS_SPI-6_ Gene Cluster

To directly link the absence of the T6SS_SPI-6_ gene cluster to the phenotype of the ΔT6SS_SPI-6_ mutant, the complete 35,921 base pair T6SS gene cluster was returned to the mutant on the self-transmissible broad-host range R995 vector. The capture of the entire T6SS_SPI-6_ gene cluster was performed using the VEX-Capture method [44] and confirmed by tiling PCR analysis (**Figure S1**).

**Figure 4:**
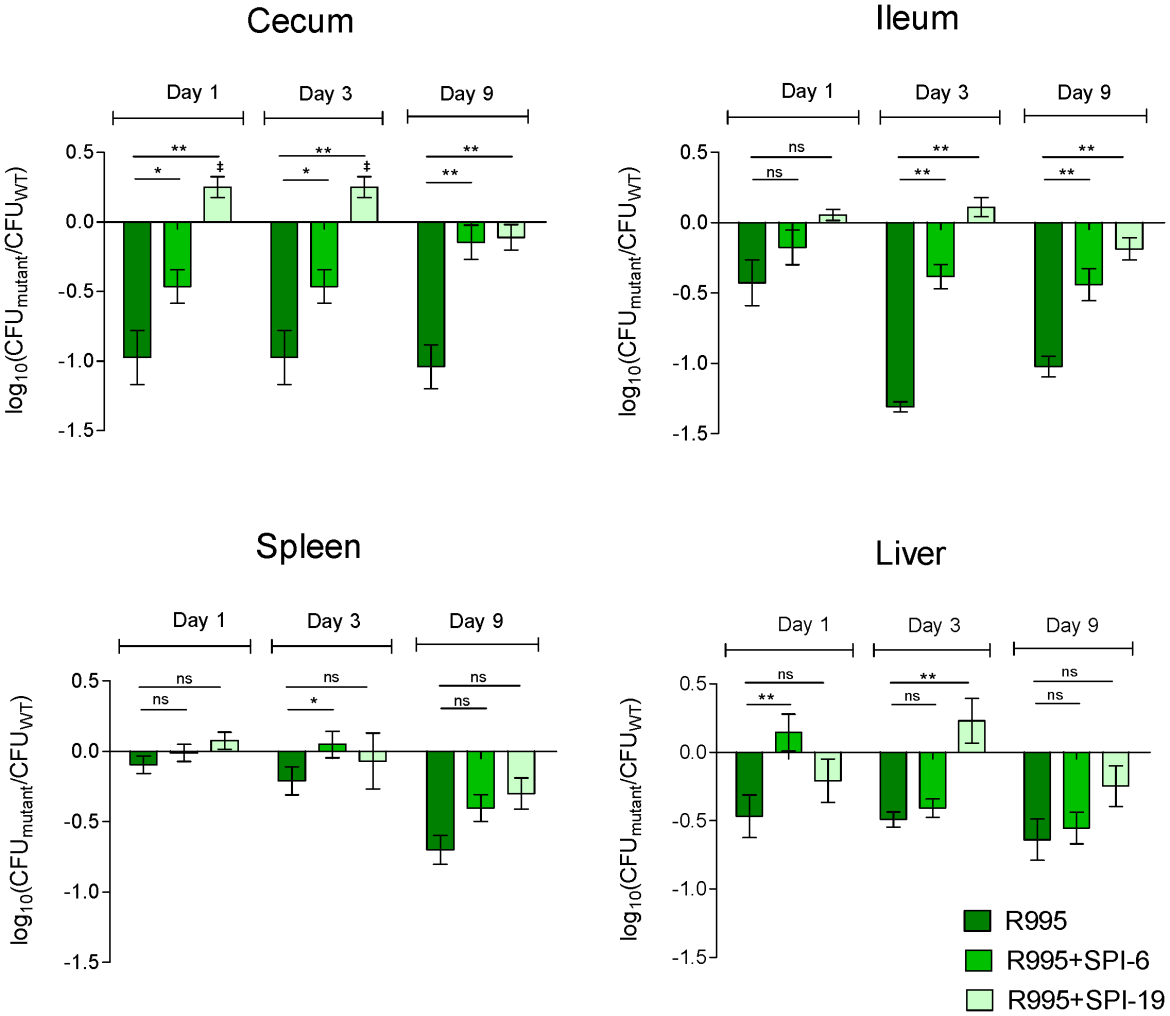
*In vivo* competition between the ΔT6SS_SPI-6_ mutants complemented *in trans* with T6SS_SPI-6_ or T6SS_SPI-19_ and the wild type *S*. Typhimurium 14028 s. Fifteen four-day-old White Leghorn chicks were orally infected with 10^9^ CFU of a mixture at a 1∶1 ratio of strains WT/R995, ΔT6SS_SPI-6_/R995+SPI-6 and ΔT6SS_SPI-6_/R995+SPI-19. At 1, 3 and 9 days post-infection groups of five chicks were sacrificed and the organs were excised, homogenized, and serially diluted for determination of bacterial loads. Bars represent the geometric mean of the log converted ratio of the mutant CFU to the wild type CFU normalized to the equivalent ratio in the inoculum. Error bars denote standard error. Statistical significance was determined using a two-tailed Student’s *t* test, and asterisks indicate statistically significant differences between normalized output ratios (*P<0.05). ^‡^Indicate statistically significant differences between normalized output ratios and the equivalent ratio in the inoculum (^‡^ *P*<0.05).

The complemented strain (MTM35R6) was tested in a competition experiment against the ΔT6SS_SPI-6_ mutant and the wild type, each bearing the empty vector (MTM35/R995 and WT/R995, respectively) and colonization was determined at days 1, 3, and 9 post infection. As shown in **Figure 4**, transfer of the T6SS_SPI-6_ gene cluster to the ΔT6SS_SPI-6_ mutant restored its ability to colonize the cecum and the ileum at all time points. On the other hand, in the spleen and liver, the results were not conclusive due to a very low and heterogeneous colonization of these deeper tissues by *S*. Typhimurium harbouring the R995 plasmid (data not shown). Nevertheless, complementation of the defective phenotype of the ΔT6SS_SPI-6_ mutant in the gastrointestinal tract supports the contribution of T6SS_SPI-6_ in chicken colonization.

### The SPI-19 T6SS from *S*. Gallinarum Restores the Colonization Defect of the SPI-6 T6SS Mutant Strain

In a previous study, we reported that T6SS_SPI-19_ contributes to efficient colonization of infected chicks by *S.* Gallinarum 287/91 [42]. T6SS_SPI-6_ and T6SS_SPI-19_ have different evolutionary histories, and were probably acquired at different times during *Salmonella* evolution [35], [36]. Because both T6SS are relevant for *Salmonella* colonization of infected chicks, we examined the possibility that both T6SS could contribute to chicken colonization in a similar extent. To test whether T6SS_SPI-19_ can restore the ability of the *S.* Typhimurium ΔT6SS_SPI-6_ mutant to efficiently colonize the avian host, the complete T6SS_SPI-19_ gene cluster captured from *S.* Gallinarum 287/91 in the R995 plasmid was transferred to *S*. Typhimurium ΔT6SS_SPI-6_ by conjugation. The resulting strain (MTM35R19) was tested in a competition experiment with the wild-type *S.* Typhimurium strain bearing the empty R995 vector (WT/R995). The results showed that introduction of the T6SS_SPI-19_ complemented the colonization defect of the ΔT6SS_SPI-6_ mutant in both the cecum and ileum (**Figure 4**). Interestingly, at days 1 and 3 post-infection, the cross-complemented strain colonized the cecum to higher levels than the wild-type strain. Analysis of the competitive fitness of the complemented strains in the spleen and liver did not show statistically significant differences; this was due to the heterogeneous and low colonization levels of systemic organs reached by Salmonellae in the chicken, as previously reported [22].

## Discussion

We previously reported that *Salmonella* encodes five distinct T6SS differentially distributed among different serotypes [35], [36]. Two of these systems, encoded in the SPI-6 and SPI-19, have been linked to the ability of serotypes Typhimurium and Gallinarum to efficiently infect mice and chickens, respectively [37], [42], [47]. Even though most of our knowledge regarding *S.* Typhimurium pathogenesis comes from murine models of infection, recent reports have highlighted the limited applicability of this model when it comes to extrapolating conclusions regarding other hosts, including the chicken.

In this work, we evaluated the contribution of T6SS_SPI-6_ to the ability of *S*. Typhimurium 14028****s to colonize the gastrointestinal tract and internal organs of White Leghorn chicks. Competitive index experiments demonstrated that the T6SS_SPI-6_ gene cluster was necessary for efficient colonization of the cecum, ileum, spleen and liver from day 1 post-infection. A similar colonization defect was observed for a mutant lacking the T6SS-essential component ClpV. Interestingly, the colonization defects were more pronounced at days 3 and 9 post-infection suggesting that mutants in the ΔT6SS_SPI-6_ do not persist well.

Histopathological analyses revealed that the attenuated phenotypes of the mutants were accompanied by changes in the inflammatory response in the cecum. Chicks infected with SPI-6 T6SS mutant strains showed considerable less inflammation and necrosis in the cecum in comparison with those infected with the wild-type strain. This could be due to the lower level of colonization of the cecum by the SPI-6 T6SS mutant compared to the wild type, or that this secretion system effectively contributes to the inflammatory response generated by *S*. Typhimurium infection. Further experiments will be needed to clarify these issues.

To confirm that T6SS_SPI-6_ was responsible for these phenotypes, the entire gene cluster was cloned and introduced in the ΔT6SS_SPI-6_ mutant. Although complementation was not observed in the spleen and liver, transfer of the T6SS gene cluster complemented the colonization defect of the mutant in the cecum and ileum throughout infection, suggesting a critical role for T6SS_SPI-6_ in the gastrointestinal phase of infection. In this context, Sivula et al. have shown that *S*. Typhimurium preferentially colonize the cecum in order to maintain a long-term persistence in chicks [22]. Therefore, T6SS_SPI-6_ may be contributing to this critical phase of the infectious process.

A role for T6SS in colonization of the gastrointestinal tract is not unexpected. Several T6SS have been linked to antibacterial killing through delivery of toxins to susceptible Gram-negative bacteria, and several authors have proposed that T6SS could contribute to bacterial adaptation and competition for new niches, including animal hosts [23]–[26], [48]. Therefore, it is possible that the defect observed in colonization of the ileum and cecum of the T6SS mutant is due to an inability of this mutant to compete with normal flora of the chicken gut. Further experiments will be needed to test this hypothesis.

On the other hand, a recent report has pointed out a role for T6SS_SPI-6_ in the intracellular survival of *S*. Typhimurium in murine macrophages [37]. Our data indicate that this secretion system is also needed for colonization of the internal organs of the chicken, suggesting a role for T6SS_SPI-6_ in intracellular survival within avian macrophages. Hence, the T6SS_SPI-6_ might contribute to both competition with the normal intestinal flora and survival within phagocytic cells.

We have previously reported that a phylogenetically distinct T6SS encoded in SPI-19, is necessary for the efficient colonization of the intestinal tract and systemic organs of chicks, and for survival of serotype Gallinarum in cultured avian macrophages [42], [49]. Because the phenotypes observed for the ΔT6SS_SPI-6_ mutant were similar to those exhibited by a ΔT6SS_SPI-19_ mutant of Gallinarum, we hypothesized that both systems could perform similar functions in chicken infection. Transfer of the T6SS_SPI-19_ gene cluster to the ΔT6SS_SPI-6_ mutant complemented the colonization defect of this strain in the ileum and cecum. Moreover, it caused an advantage for colonization of cecum at days 1 and 3 post-infection. These results indicate that both T6SS, despite their different evolutionary histories, contribute to a similar extent to chicken colonization by *Salmonella*. This statement is supported by the fact that both SPI-6 and SPI-19 T6SS have been shown to be required for *Salmonella* intracellular survival within macrophages [37], [49].

Altogether, we have determined that T6SS_SPI-6_ contributes to chicken colonization by *S.* Typhimurium. Also, we show that T6SS_SPI-19_ from the avian-adapted serotype Gallinarum is able to replace T6SS_SPI-6_, suggesting a broad role for these secretion systems in *Salmonella* host colonization. Most interestingly, our results indicate that T6SS_SPI-19_ confers an advantage to *S.* Typhimurium to colonize the gastrointestinal tract of the chicks early in infection.

## Supporting Information

Figure S1 ***In vivo*** **cloning of T6SS_SPI-6_ from** ***S*** **. Typhimurium 14028 s.** **(A)** Scheme of the VEX-Capture procedure: *loxP* sites were inserted in the chromosome of *S*. Typhimurium 14028****s at each side of the T6SS_SPI-6_ gene cluster through homologous recombination of PCR products using the Lambda-Red system. In presence of pEKA30, a plasmid that constitutively expresses the Cre recombinase, the T6SS cluster was excised from the chromosome as a non-replicative, circular DNA intermediate that was captured through homologous recombination in R995-VC6, a derivative of R995 plasmid harboring an internal region of homology to T6SS_SPI-6_. **(B)** Tiling-PCR analysis of the T6SS_SPI-6_ gene cluster cloned onto the R995 plasmid. Specific primers were designed to amplify ten fragments that cover the entire T6SS_SPI-6_ region and whose lengths vary between 3,298 and 4,274 bp.
(TIF)
