# Supplementary figures and images for: The Type VI Secretion System Encoded in SPI-6 Plays a Role in Gastrointestinal Colonization and Systemic Spread of Salmonella enterica serovar Typhimurium in the Chicken
Source: PLoS One. 2013 May 14;8(5):e63917. doi: 10.1371/journal.pone.0063917 (PMC3653874; doi:10.1371/journal.pone.0063917)

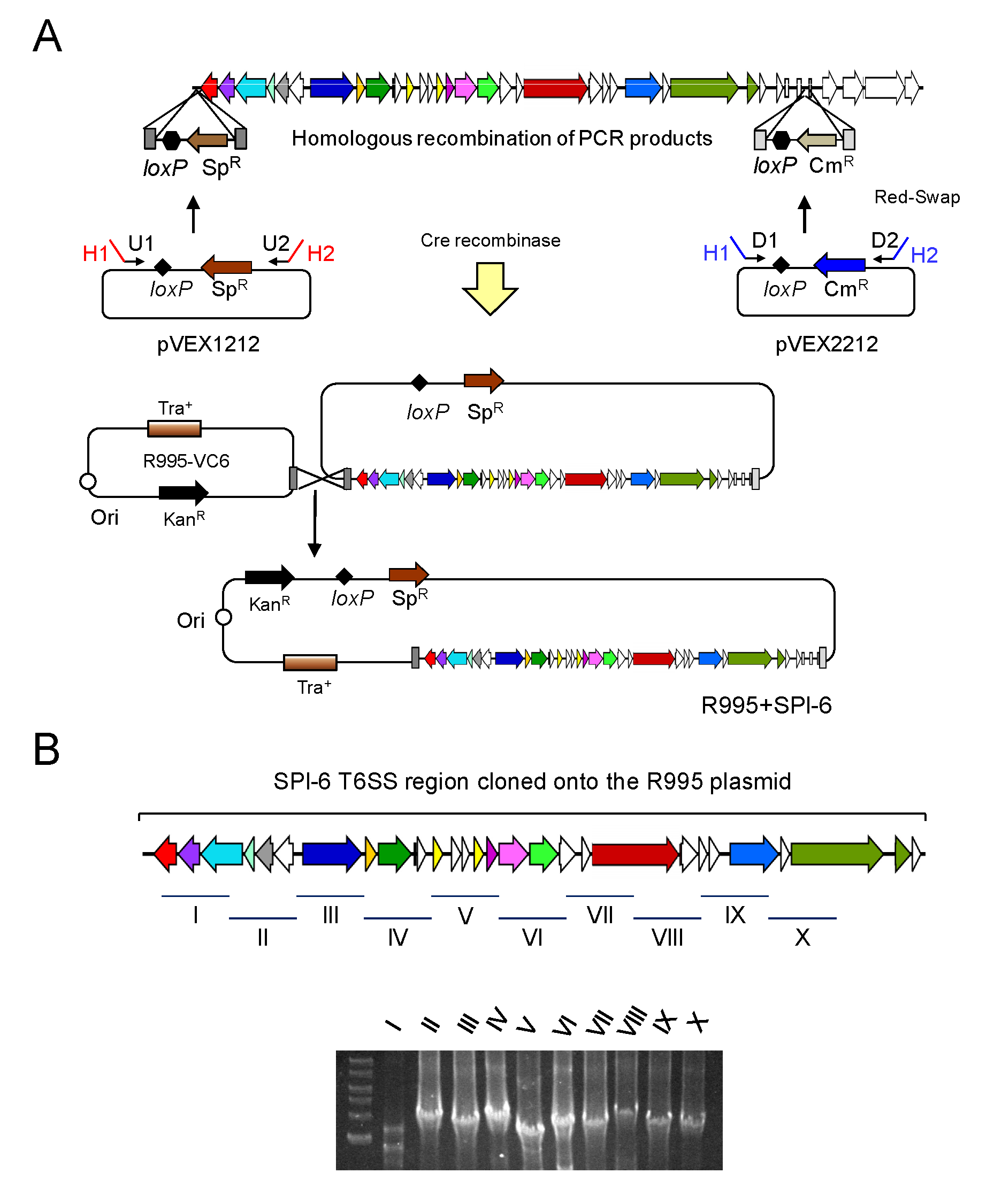

Supplement: Figure S1 — In vivo cloning of T6SSSPI-6 from S . Typhimurium 14028 s. (A) Scheme of the VEX-Capture procedure: loxP sites were inserted in the chromosome of S. Typhimurium 14028s at each side of the T6SSSPI-6 gene cluster through homologous recombination of PCR products using the Lambda-Red system. In presence of pEKA30, a plasmid that constitutively expresses the Cre recombinase, the T6SS cluster was excised from the chromosome as a non-replicative, circular DNA intermediate that was captured through homologous recombination in R995-VC6, a derivative of R995 plasmid harboring an internal region of homology to T6SSSPI-6. (B) Tiling-PCR analysis of the T6SSSPI-6 gene cluster cloned onto the R995 plasmid. Specific primers were designed to amplify ten fragments that cover the entire T6SSSPI-6 region and whose lengths vary between 3,298 and 4,274 bp. (TIF) [file pone.0063917.s001.tif]
